# Association between caffeine intake and lumbar spine bone mineral density in adults aged 20–49: A cross-sectional study

**DOI:** 10.3389/fendo.2022.1008275

**Published:** 2022-10-17

**Authors:** Gaoxiang Wang, Ze-Bin Fang, De-Liang Liu, Shu-Fang Chu, Hui-Lin Li, Heng-Xia Zhao

**Affiliations:** ^1^ Department of Endocrinology, Shenzhen Traditional Chinese Medicine Hospital Affiliated to Nanjing University of Chinese Medicine, Shenzhen, China; ^2^ Department of Endocrinology, Shenzhen Traditional Chinese Medicine Hospital, Shenzhen, China; ^3^ The Fourth Clinical Medical College of Guangzhou University of Chinese Medicine, Shenzhen, China

**Keywords:** caffeine intake, bone mineral density, NHANES, cross-sectional study, osteoporosis

## Abstract

**Background:**

Many epidemiological studies have investigated the connection between coffee intake and bone mineral density (BMD), but the results are controversial. This study aimed to assess the association between caffeine consumption and lumbar BMD in adults aged 20–49.

**Methods:**

From a cross-sectional study based on a large sample of the National Health and Nutrition Examination Survey 2011–2018. After controlling for confounders, the weighted multivariate linear regression model was created and stratified by age, gender, and race for subgroup analysis. In addition, we simultaneously stratified analysis by age and sex and divided caffeine intake into quartiles to assess the association between coffee intake and BMD.

**Results:**

Caffeine intake was not significantly linked with lumbar BMD in this study of 7041 adults. In subgroup studies stratified by age, there was a significant correlation between lumbar BMD and caffeine consumption in participants aged 30–39 and 40–49. In females, there was a positive correlation between lumbar BMD and coffee consumption stratified by gender. When evaluated by race, the association between lumbar BMD and caffeine intake was independent of race. Consequently, when stratifying for age, sex, and coffee intake quartiles, a significant positive correlation was discovered between the fourth coffee intake quartile and lumbar BMD in females aged 30–39. In addition, a negative correlation was discovered between coffee consumption and lumbar BMD in males aged 40–49.

**Conclusions:**

Our research indicates that drinking coffee may benefit 30–39 women’s lumbar BMD, but it may adversely affect men aged 40–49.

## Introduction

Osteoporosis (OP) is a degenerative disease of the bones that results in weakened bones, weakened microarchitecture, increased fragility, and increased fracture risk (1, 2). Owing to the development of an aging population, osteoporosis has become the most common bone-related chronic disease and the bone metabolic disease with the highest incidence. According to a worldwide survey by the International Society for Clinical Densitometry and the International Foundation for Osteoporosis, more than 70 million Americans will be diagnosed with osteoporosis or bone loss by 2030 (3). The economic burden of osteoporosis-related fractures is significant, costing approximately $17.9 billion annually in the United States (4). Therefore, clinical attention should be focused on identifying modifiable osteoporosis risk factors, such as coffee drinking.

Coffee is one of the most widely consumed beverages in the world nowadays. According to a survey conducted by the National Coffee Association, roughly 64% of adults in the United States drink coffee daily, and approximately 517 million cups of coffee are consumed daily (5). Therefore, researchers pay more attention to the effect of caffeine on human health. Epidemiological research (6–11) indicates that coffee consumption can prevent or decrease the risk of cardiovascular disease, chronic liver conditions, neurodegeneration, and cancer. However, coffee consumption can also adversely affect the human body, such as sleep disturbance, anxiety, and poor pregnancy outcomes (12). Coffee can significantly affect metabolic levels in adults and has been proven in numerous studies to affect metabolic diseases such as obesity and diabetes significantly (13–16).

In the past, a great deal of epidemiological research has been carried out in order to investigate the connection between coffee consumption and BMD. However, the conclusions have been inconsistent. In a Taiwanese longitudinal study, Chang et al. found drinking coffee to have a significantly beneficial relationship with BMD in both males and premenopausal women (17). However, Hallstrom et al. observed that high coffee intake in middle-aged and elderly Swedish women decreased BMD by 2%–4% compared to low coffee intake (18). It is puzzling that Demirbag et al. investigated 200 premenopausal individuals and found no correlation between coffee consumption and BMD (19). These contradictory results may be attributable to their demographic traits, small sample size, bias in data collection, and other aspects. Therefore, we used the National Health and Nutrition Examination Survey (NHANES) database from 2011 to 2018 to conduct a large-scale, broadly representative clinical study on the connection between coffee consumption and BMD to guide clinicians.

## Methods

### Data source and study population

This study is based on 2011–2018 NHANES data. The NHANES is a nationally representative sample database that assesses the physical and mental well-being of adults and children in the United States. The NHANES has a complicated, multi-stage stratified sample design that covers individuals from various life backgrounds and is representative of the population as a whole. In addition to demographic, socioeconomic, and nutritional questions, the survey also includes physiological measurements and laboratory tests. The information collected by NHANES is used by public health officials, legislators, and clinicians to estimate how common chronic diseases are and to create good public health policies, public health initiatives, and services that protect the health of the population.

**Figure 1:**
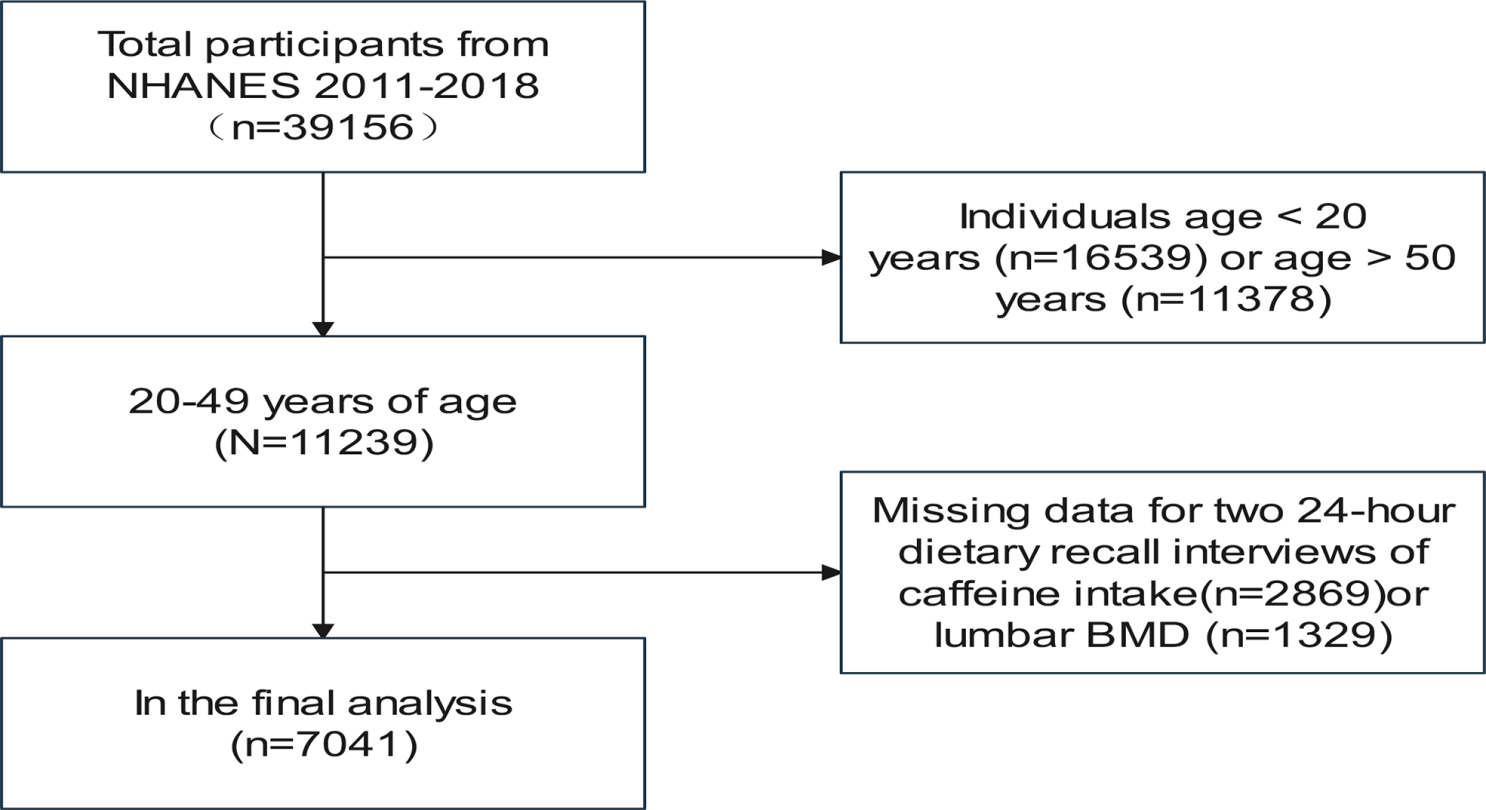
Flowchart of participants selection.

We retrieved a total of 39,156 participants from the 2011–2018 NHANES database, and we excluded 11378 subjects older than 49 years and 16,539 subjects younger than 20. Ultimately, 7041 participants were included in our investigation after we eliminated 5493 adults who had missed two 24-hour dietary recall interviews about caffeine use (n = 2869) or lumbar BMD (n = 1329)(**Figure 1**).

### Ethics statement

NHANES requires all survey participants to sign an informed consent form, which is evaluated and authorized by the National Center for Health Statistics Ethics Review Board. After privacy has been protected, the data are now available to the public. It is already feasible to convert data into an analyzed format. All statistics will be used for research methodology, and all research will comply with applicable laws and standards as long as we follow the research’s data usage rules.

### Covariates

In this study, we used two 24–hour dietary recall interviews to determine how much caffeine each participant drank daily. The first 24-hour dietary recall interview was conducted in person at the Mobile Examination Center (MEC), and the second 24–hour dietary recall interview was conducted by phone 3–10 days after the MEC. All participants were required to complete both interviews. The subjects were evaluated using dual-energy X-ray absorptiometry (DXA), performed on a Hologic Discovery Model A densitometer (Hologic, Inc., Bedford, Massachusetts) using Apex version 3.2 software. The radiographer who performed the evaluations was trained and certified. A whole-body scan was performed, and the subject’s BMD was calculated (Containing lumbar spine BMD information but no femoral neck BMD). In the Body Composition Procedure Manual, which may be found on the NHANES website, there is further information regarding the DXA examination protocol. In addition, we incorporated gender, race/ethnicity, educational level, and moderate exercise as categorical variables in this study. Age, BMI, the ratio of family income to poverty, alkaline phosphatase, blood calcium, blood phosphorus, blood uric acid, total cholesterol, triglyceride, glycohemoglobin, blood urea nitrogen, serum creatinine, urinary albumin creatinine ratio, total albumin, average caffeine intake, lumbar BMD are continuous variables. Visit www.cdc.gov/nchs/nhanes/ to learn more about how covariate data were collected, how two 24-hour recall interviews were conducted, and how lumbar BMD outcome variables were measured.

### Statistical analyses

All of the estimates in this investigation were computed using sample weights, following recommended by the NCHS. Continuous variables are shown as the Mean ± SD, and the weighted multiple linear regression model was utilized to determine whether there were differences between the various groupings of continuous variables. The chi-square test results were analyzed to see whether or not there was a significant difference between the categorical variable components. The results of the test are reported as %. Following the recommendations provided by Reporting on Observational Studies in Strengthening Epidemiology, three weighted multivariate linear regression models were created to acquire a more comprehensive understanding of the association between coffee consumption and BMD. Model 1 was left untouched. Model 2 has been tweaked to consider age, gender, and race/ethnicity. Model 3 adjusted for all confounders. We did subgroup analyses based on age, gender, and race to get the most out of these data and investigate further the association between coffee consumption and lumbar BMD. The EmpowerStats (http://www.empowerstats.com) and R (http://www.r-project.org) were utilized for every statistical analysis. In general, we regarded *P* < 0.05 as statistically meaningful.

## Results

**Table 1** provides the weighted characteristics of the study samples, which include demographic and medical characteristics. The research included 7041 subjects, with 3565 males and 3476 females participating. The average age of men was 33.65 ± 8.62 years old, and that of women was 34.42 ± 8.84 years old, and the difference was statistically meaningful. The daily caffeine intake of male participants was 0.15 ± 0.18 g, and that of female participants was 0.13 ± 0.15 g, and the difference was also statistically meaningful. Furthermore, the difference still existed in educational level, ratio of family income to poverty, moderate activity, alkaline phosphatase, blood calcium, total cholesterol, blood uric acid, triglyceride, blood urea nitrogen, blood creatinine, glycohemoglobin, lumbar BMD and total protein. No statistically significant differences were observed between the male and female participants in terms of,race or ethnicity, BMI, blood phosphorus, and the urine albumin creatinine to ratio.

**Table 1:** Weighted characteristics of the study sample.

	Male (n=3565)	Female (n=3476)	*P* value
**Age (years)**	33.65 ± 8.62	34.42 ± 8.84	< 0.001
**Race/ethnicity (%)**			0.261
White	57.74	58.06	
Black	11.6	12.89	
Mexican American	12.4	11.61	
Other race	18.25	17.44	
**Educational level, n (%)**			< 0.001
Less than high school	12.6	11.37	
High school	23.4	17.96	
More than high school	64	70.67	
**Ratio of family income to poverty (%)**	2.90 ± 1.62	2.72 ± 1.61	< 0.001
**Moderate activities (%)**			<0.001
**No**	25.87	29.9	
**Yes**	74.13	70.1	
**Body mass index (kg/m^2^)**	28.72 ± 6.22	28.99 ± 7.62	0.104
**Alkaline phosphatase (u/l)**	66.96 ± 21.76	63.42 ± 21.16	< 0.001
**Serum calcium (mmol/l)**	2.37 ± 0.08	2.32 ± 0.08	< 0.001
**Serum phosphorus (mmol/l)**	1.20 ± 0.19	1.21 ± 0.17	0.054
**Serum uric acid (umol/l)**	361.15 ± 71.95	272.74 ± 61.75	< 0.001
**Total cholesterol (mmol/l)**	4.92 ± 1.02	4.79 ± 0.90	< 0.001
**Triglyceride (mmol/l)**	1.93 ± 1.72	1.40 ± 1.20	< 0.001
**Glycohemoglobin (%)**	5.41 ± 0.77	5.37 ± 0.71	0.026
**Blood urea nitrogen (mmol/l)**	4.80 ± 1.39	4.04 ± 1.33	< 0.001
**Serum creatinine (umol/l)**	85.06 ± 16.67	65.22 ± 20.21	< 0.001
**Urinary albumin creatinine ratio (mg/g)**	18.81 ± 215.08	23.62 ± 123.91	0.257
**Total protein (g/l)**	72.30 ± 4.14	71.21 ± 4.06	< 0.001
**Average caffeine intake (g/day)**	0.15 ± 0.18	0.13 ± 0.15	< 0.001
**Lumbar bone mineral density (g/cm^2^)**	1.03 ± 0.15	1.06 ± 0.14	< 0.001

Continuous variables are presented as Mean ± SD, P-value was calculated by a weighted linear regression model. Categorical variables are presented as %, P-value was calculated by chi-square test.

**Table 2** presents three weighted multivariate linear regression models. In Model 1, without adjustment for variables, there was a statistically negative association between caffeine consumption and lumbar BMD. As for Model 2 with partial adjustment for covariates and model 3 with adjustment for all variables, average caffeine intake and lumbar BMD were linked, but the link was not statistically significant. When stratified by age, average caffeine consumption was positively and significantly associated with lumbar BMD in adults aged 30–39 and 40–49. Average caffeine intake in female adults was significantly positively associated with lumbar BMD when stratified by gender. Coffee intake and lumbar BMD were not associated with race when evaluated by race.

**Table:** Table 2 Association between average caffeine intake (g/day) and lumbar bone mineral density (g/cm^2^).

Exposure	Model 1, β (95% CI)	Model 2, β (95% Cl)	Model 3, β (95% CI)
**Average caffeine intake (g/day)**	-0.027 (-0.048, -0.007)**	-0.002 (-0.023, 0.019)	0.000 (-0.021, 0.021)
**Stratified by age**			
20**–**29 years old	-0.015 (-0.057, 0.028)	0.016 (-0.027, 0.058)	0.022 (-0.020, 0.064)
30**–**39 years old	0.009 (-0.028, 0.046)*	0.040 (0.002, 0.077)	0.040 (0.002, 0.077)*
40**–**49 years old	-0.054 (-0.086, -0.022)**	-0.035 (-0.068, -0.003)*	-0.033 (-0.065, -0.001)*
**Stratified by gender**			
Male	-0.055 (-0.082, -0.028)***	-0.022 (-0.050, 0.006)	-0.019 (-0.046, 0.009)
Female	0.036 (0.005, 0.068)*	0.039 (0.005, 0.072)*	0.037 (0.004, 0.070)*
**Stratified by race**			
White	-0.004 (-0.035, 0.027)	0.006 (-0.026, 0.038)	0.009 (-0.022, 0.040)
Black	0.026 (-0.063, 0.115)	0.016 (-0.075, 0.107)	0.010 (-0.081, 0.100)
Mexican American	-0.066 (-0.141, 0.009)	-0.027 (-0.104, 0.051)	-0.038 (-0.117, 0.041)
Other race	-0.026 (-0.064, 0.012)	-0.021 (-0.059, 0.017)	-0.024 (-0.062, 0.014)

Model 1: All variables were not adjusted.

Model 2: Age, sex, race were adjusted.

Model 3: All variables were adjusted.

The model is not adjusted for the stratification variable itself in the subgroup analysis.

*P<0.05, **P<0.01, ***P<0.001.

**Table 3** displays the correlation between average caffeine consumption and BMD stratified by age and sex. Caffeine consumption was found to be positively related to lumbar BMD in females aged 30–39 and negatively related to lumbar BMD in males aged 40–49.By further stratifying the average caffeine intake, we studied the relationship between caffeine intake and lumbar bone mineral density and took the lowest quartile of caffeine intake as the control group. The trend between the quartile of average caffeine intake and lumbar BMD remained significant in the two subgroups.Notably, in the subgroup of women aged 30 – 39 years, the fourth quartile was significantly different from the lowest group, but no difference was found among the four quartile in the subgroup of men aged 40 – 49 years.

**Table 3:** Association between Average caffeine intake (g/day) and lumbar bone mineral density (g/cm^2^), stratified by age and gender.

Quintiles of average caffeine intake (g/day)	Lumbar bone mineral density (g/cm^2^), β (95% Cl)	
	Male	Female
**20–29 years old**	0.025 (-0.029, 0.078)	0.056 (-0.012, 0.125)
Lowest quartiles	reference	reference
2^nd^	0.004 (-0.017, 0.025)	-0.007 (-0.026, 0.012)
3^rd^	-0.003 (-0.024, 0.019)	0.006 (-0.013, 0.026)
4^th^	-0.019 (-0.042, 0.003)	0.014 (-0.008, 0.036)
*P* for trend	0.096	0.158
**30–39 years old**	0.023 (-0.024, 0.071)	0.080 (0.019, 0.142)*
Lowest quartiles	reference	reference
2^nd^	-0.006 (-0.033, 0.020)	0.009 (-0.014, 0.033)
3^rd^	-0.012 (-0.037, 0.012)	0.012 (-0.013, 0.036)
4^th^	0.003 (-0.020, 0.026)	0.025 (0.000, 0.050)*
*P* for trend	0.748	0.049
**40–49 years old**	-0.070 (-0.113, -0.026)**	0.007 (-0.041, 0.054)
Lowest quartiles	reference	reference
2^nd^	0.001 (-0.032, 0.033)	0.013 (-0.015, 0.040)
3^rd^	0.016 (-0.018, 0.049)	0.020 (-0.007, 0.047)
4^th^	-0.027 (-0.058, 0.004)	0.011 (-0.016, 0.037)
*P* for trend	0.026	0.553

All variables were adjusted.

*P<0.05, **P<0.01.

## Discussion

Coffee is the most popular beverage in the world. Osteoporosis is a common endocrine and metabolic disease. BMD is one of the most important diagnostic indexes of OP. Many epidemiological studies have been conducted to investigate the connection between coffee consumption and BMD, but the conclusions are controversial (17, 18, 20). As a result, we used the NHANES database to perform this extensive, representative cross-sectional study.

From the NHANES 2011–2018, we chose a representative sample of 9041 adults aged 20–49. In subgroups stratified by age, according to our data, a significant correlation existed between the consumption of coffee and lumbar BMD in individuals aged 30–39 and 40–49. In subgroups stratified by gender, we found that association between caffeine intake and bone mineral density is also influenced by gender. The three models failed to find this connection in subgroups stratified by race. Since the association between the amount of caffeine consumed and the BMD of the lumbar spine varies depending on age and gender, we further analyzed and stratified gender and age simultaneously. We found that women aged 30–39 had a significant positive association between coffee consumption and BMD and that in this group. However, in 40–49 years old men subgroup, we found a negative correlation.

The effect of coffee consumption on BMD is controversial as one of the three most popular beverages in the world. In a clinical study of 4066 postmenopausal women, Choi et al. analyzed by multivariate logistic regression, adjusting for all confounding factors, that the highest coffee intake group had a 36% lower prevalence of osteoporosis than the lowest group (*P* = 0.015) (21). In a prospective study with up to 30 years of follow-up, researchers randomly selected 7495 men aged 46–56 and found that coffee consumption reduced the incidence of hip fractures. Researchers have found that males who drink coffee have a lower risk of fractures (22). Similarly, Yu et al. demonstrated in a large, community-based cross-sectional study that moderate coffee consumption decreases the prevalence of osteoporosis in men (23). In a recent meta-analysis of approximately 400,000 participants, researchers found that people with high coffee intake had a lower risk of osteoporosis than those with low coffee intake [OR (95% CI): 0.79 (0.65–0.92)] (24). A Hong Kong OP study showed that the serum metabolite levels of caffeine were significantly correlated with BMD, which to some extent, confirmed our findings (25). As for the potential pathophysiological aspects of the effect of coffee consumption on BMD. In a recent study, Berman et al. showed that caffeine regulates osteoblast/osteoclast differentiation through nonspecific antagonism of adenosine receptors (26). Ankita et al. suggested that the physiological release of purine in the extracellular space plays an important role in bone homeostasis, both by acting on membrane receptors directly affecting osteocytes and by synergistic stimulation with some osteoactive hormones (27). In addition, researchers also believe that caffeine affects bone metabolism through mechanisms such as regulating calcium and altering the lipid profile (28). Consistent with previous studies, we believe that young women who consume coffee can improve their lumbar spine BMD. However, the finding that men aged 40–49 years who drink coffee may have a potential risk of lumbar spine BMD contradicts previous studies, and we did not find an association after stratifying all variables only by sex, so the conclusions of this male-specific study need to be cautiously interpreted.

Nevertheless, there are some concerns with our study. People older than 50 years were not included in this study due to a scarcity of data, resulting in these subjects being excluded from the analysis, which severely limits the possibility of considering the basic endpoint of fragility fracture risk when referring to “bone health.” This is one of the major limitations of this study. Because this was a cross-sectional study, it was not possible to determine whether or not there was a causal connection between the use of coffee and lumbar spine BMD. Due to the limitations of the questionnaire, personal eating habits and lifestyles could not be assessed, and the menstrual conditions, sex hormone levels, and drug use of female participants may have affected the study’s findings. Levels of bioactive were unknown. The specific mechanism of coffee action is not involved in this study. This study has many covariates, and there may be some collinearity between them. In addition, hip femoral neck BMD was not included in this study due to data limitations. Despite these limitations, it is undeniable that our study’s sample size is significant and that it represents a sophisticated, stratified, multi-stage probabilistic sample of the non-institutionalized American population. In terms of survey techniques and quality assurance, NHANES is of high caliber. Additionally, after adjusting for various confounders, we performed weighted multiple linear regression analysis and subgroup analyses stratified by age, gender, and ethnicity to assess the effect of coffee consumption on BMD. Most importantly, the findings of this study provide new information for patients with osteoporosis or low bone mass since they demonstrate that drinking coffee has a beneficial impact on BMD in young women. For future research on this topic, due to the lack of data on people over 50 years of age in this study, we suggest that future studies take more into account factors such as menopause and old age. Considering the limitations and shortcomings of our study, we recommend that more prospective studies be conducted in the future to confirm the causal relationship between caffeine and BMD. In addition, coffee contains various active ingredients, and future studies should fully consider the effects of other minerals and other factors on bone.

In conclusion, according to this study, drinking coffee may benefit women aged 30–39 lumbar BMD, but may negatively affect men aged 40–49. Although our study is focused on caffeine, in our daily life, we also need to pay attention to the differences in the content of minerals, lipids, proteins, and other substances in different coffee powders, which may also have an impact on bone health, although it is difficult to weight them because they are not easy to calculate and consider in observational studies.

## Data availability statement

Publicly available datasets were analyzed in this study. The survey data can be found here: www.cdc.gov/nchs/nhanes/.

## Ethics statement

NHANES protocols were approved by the Research Ethics Review Board of NCHS, and Written informed consent was obtained from each participant.

## Author contributions

All authors listed have made a substantial, direct, and intellectual contribution to the work and approved it for publication.

## Funding

This study was funded by the The Shenzhen Municipal Science and Technology Bureau (No. JCYJ20170817094838619 & JCYJ20180302173821841); National Natural Science Foundation of China (No. 82104759); and The Natural Science Foundation of Guangdong Provincial (No. 2019A1515110108).

## Conflict of interest

The authors declare that the research was conducted in the absence of any commercial or financial relationships that could be construed as a potential conflict of interest.

## Publisher’s note

All claims expressed in this article are solely those of the authors and do not necessarily represent those of their affiliated organizations, or those of the publisher, the editors and the reviewers. Any product that may be evaluated in this article, or claim that may be made by its manufacturer, is not guaranteed or endorsed by the publisher.

## References

[1] Gregson CL Armstrong DJ Bowden J Cooper C Edwards J Gittoes N. Correction: UK clinical guideline for the prevention and treatment of osteoporosis. Arch Osteoporos (2022) 17:80. doi: 10.1007/s11657-022-01115-8 35585444 PMC9117369

[2] Gonzalez RE Debrach-Schneider AC Lamy O. [Osteoporosis]. Rev Med Suisse (2022) 18:56–8. doi: 10.53738/REVMED.2022.18.764-65.56 35048581

[3] Clynes MA Westbury LD Dennison EM Kanis JA Javaid MK Harvey NC. Bone densitometry worldwide: A global survey by the ISCD and IOF. Osteoporos Int (2020) 31:1779–86. doi: 10.1007/s00198-020-05435-8 PMC7115939 32377806

[4] Clynes MA Harvey NC Curtis EM Fuggle NR Dennison EM Cooper C. The epidemiology of osteoporosis. Br Med Bull (2020) 133:105–17. doi: 10.1093/bmb/ldaa005 PMC7115830 32282039

[5] Feng J Wang J Jose M Seo Y Feng L Ge S. Association between caffeine intake and all-cause and cause-specific mortality: An analysis of the national health and nutrition examination survey (NHANES) 1999-2014 database. Nurs Rep (2021) 11:901–12. doi: 10.3390/nursrep11040083 PMC8715461 34968277

[6] Mansour A Mohajeri-Tehrani MR Samadi M Qorbani M Merat S Adibi H. Effects of supplementation with main coffee components including caffeine and/or chlorogenic acid on hepatic, metabolic, and inflammatory indices in patients with non-alcoholic fatty liver disease and type 2 diabetes: A randomized, double-blind, placebo-controlled, clinical trial. Nutr J (2021) 20:35. doi: 10.1186/s12937-021-00694-5 33838673 PMC8037901

[7] Sartini M Bragazzi NL Spagnolo AM Schinca E Ottria G Dupont C. Coffee consumption and risk of colorectal cancer: A systematic review and meta-analysis of prospective studies. Nutrients (2019) 11:694. doi: 10.3390/nu11030694 PMC6471028 30909640

[8] Wijarnpreecha K Thongprayoon C Ungprasert P. Coffee consumption and risk of nonalcoholic fatty liver disease: A systematic review and meta-analysis. Eur J Gastroenterol Hepatol (2017) 29:e8–e12. doi: 10.1097/MEG.0000000000000776 27824642

[9] Grioni S Agnoli C Sieri S Pala V Ricceri F Masala G. Espresso coffee consumption and risk of coronary heart disease in a large Italian cohort. PloS One (2015) 10:e0126550. doi: 10.1371/journal.pone.0126550 25946046 PMC4422699

[10] Wu L Sun D He Y. Coffee intake and the incident risk of cognitive disorders: A dose-response meta-analysis of nine prospective cohort studies. Clin Nutr (2017) 36:730–6. doi: 10.1016/j.clnu.2016.05.015 27288328

[11] Liu D Li ZH Shen D Zhang PD Song WQ Zhang WT. Association of sugar-sweetened, artificially sweetened, and unsweetened coffee consumption with all-cause and cause-specific Mortality: A Large prospective cohort study. Ann Intern Med (2022) 175:909–17. doi: 10.7326/M21-2977 35635846

[12] Wikoff D Welsh BT Henderson R Brorby GP Britt J Myers E. Systematic review of the potential adverse effects of caffeine consumption in healthy adults, pregnant women, adolescents, and children. Food Chem Toxicol (2017) 109:585–648. doi: 10.1016/j.fct.2017.04.002 28438661

[13] Harty PS Stratton MT Escalante G Rodriguez C Dellinger JR Williams AD. Effects of bang® keto coffee energy drink on metabolism and exercise performance in resistance-trained adults: A randomized, double-blind, placebo-controlled, crossover study. J Int Soc Sports Nutr (2020) 17:45. doi: 10.1186/s12970-020-00374-5 32831109 PMC7446127

[14] Pechmann LM Petterle RR Moreira CA Borba VZC. Osteosarcopenia and trabecular bone score in patients with type 2 diabetes mellitus. Arch Endocrinol Metab (2021) 65:801–10. doi: 10.20945/2359-3997000000418 PMC10065394 34762788

[15] Lee CY Back GY Lee SH. Relationship between type 2 diabetes mellitus and lumbar bone mineral density in postmenopausal women. Asian Spine J (2021) 15:721–7. doi: 10.31616/asj.2021.0099 PMC8696060 34551503

[16] Cherukuri L Kinninger A Birudaraju D Lakshmanan S Li D Flores F. Effect of body mass index on bone mineral density is age-specific. Nutrition Metab Cardiovasc Dis (2021) 31:1767–73. doi: 10.1016/j.numecd.2021.02.027 33934946

[17] Chang HC Hsieh CF Lin YC Tantoh DM Ko PC Kung YY. Does coffee drinking have beneficial effects on bone health of Taiwanese adults? a longitudinal study. BMC Public Health (2018) 18:1273. doi: 10.1186/s12889-018-6168-0 30453911 PMC6245613

[18] Hallström H Byberg L Glynn A Lemming EW Wolk A Michaëlsson K. Long-term coffee consumption in relation to fracture risk and bone mineral density in women. Am J Epidemiol (2013) 178:898–909. doi: 10.1093/aje/kwt062 23880351

[19] Demirbag D Ozdemir F Ture M. Effects of coffee consumption and smoking habit on bone mineral density. Rheumatol Int (2006) 26:530–5. doi: 10.1007/s00296-005-0020-4 16025331

[20] Rychter AM Ratajczak AE Szymczak-Tomczak A Michalak M Eder P Dobrowolska A. Associations of lifestyle factors with osteopenia and osteoporosis in polish patients with inflammatory bowel disease. Nutrients (2021) 13:1863. doi: 10.3390/nu13061863 34070791 PMC8227497

[21] Choi E Choi KH Park SM Shin D Joh HK Cho E. The benefit of bone health by drinking coffee among Korean postmenopausal women: A cross-sectional analysis of the fourth & fifth Korea national health and nutrition examination surveys. PloS One (2016) 11:e0147762. doi: 10.1371/journal.pone.0147762 26816211 PMC4729688

[22] Trimpou P Landin-Wilhelmsen K Odén A Rosengren A Wilhelmsen L. Male Risk factors for hip fracture-a 30-year follow-up study in 7,495 men. Osteoporos Int (2010) 21:409–16. doi: 10.1007/s00198-009-0961-7 19475474

[23] Yu Q Liu ZH Lei T Tang Z. Subjective evaluation of the frequency of coffee intake and relationship to osteoporosis in Chinese men. J Health Popul Nutr (2016) 35:24. doi: 10.1186/s41043-016-0060-2 27495290 PMC5026020

[24] Zeng X Su Y Tan A Zou L Zha W Yi S. The association of coffee consumption with the risk of osteoporosis and fractures: A systematic review and meta-analysis. Osteoporos Int (2022) 33:1871–93. doi: 10.1007/s00198-022-06399-7 35426508

[25] Chau Y-P Au PCM Li GHY Sing C-W Cheng VKF Tan KCB. Serum metabolome of coffee consumption and its association with bone mineral density: The Hong Kong osteoporosis study. J Clin Endocrinol Metab (2020) 105:dgz210. doi: 10.1210/clinem/dgz210 31750515

[26] Berman NK Honig S Cronstein BN Pillinger MH. The effects of caffeine on bone mineral density and fracture risk. Osteoporos Int (2022) 33:1235–41. doi: 10.1007/s00198-021-05972-w 34981132

[27] Agrawal A Jørgensen NR. Extracellular purines and bone homeostasis. Biochem Pharmacol (2021) 187:114425. doi: 10.1016/j.bcp.2021.114425 33482152

[28] Xu H Liu T Hu L Li J Gan C Xu J. Effect of caffeine on ovariectomy-induced osteoporosis in rats. Biomed Pharmacother (2019) 112:108650. doi: 10.1016/j.biopha.2019.108650 30797144

